# Enhanced regeneration and functional recovery after spinal root avulsion by manipulation of the proteoglycan receptor PTPσ

**DOI:** 10.1038/srep14923

**Published:** 2015-10-14

**Authors:** Heng Li, Connie Wong, Wen Li, Carolin Ruven, Liumin He, Xiaoli Wu, Bradley T. Lang, Jerry Silver, Wutian Wu

**Affiliations:** 1 School of Biomedical Sciences, Division of Anatomy, the University of Hong Kong, 21 Sassoon Road, Pokfulam, Hong Kong SAR, China; 2 State Key Laboratory of Brain and Cognitive Sciences, Li Ka Shing Faculty of Medicine, The University of Hong Kong, Pokfulam, Hong Kong SAR, China; 3 GHM Institute of CNS Regeneration, Jinan University, Guangzhou, China; 4 Department of Neurosciences, Case Western Reserve University School of Medicine, 2109 Adelbert Road Cleveland, OH 44106, USA

## Abstract

Following root avulsion, spinal nerves are physically disconnected from the spinal cord. Severe motoneuron death and inefficient axon regeneration often result in devastating motor dysfunction. Newly formed axons need to extend through inhibitory scar tissue at the CNS-PNS transitional zone before entering into a pro-regenerative peripheral nerve trajectory. CSPGs are dominant suppressors in scar tissue and exert inhibition via neuronal receptors including PTPσ. Previously, a small peptide memetic of the PTPσ wedge region named ISP (Intracellular Sigma Peptide) was generated, and its capabilities to target PTPσ and relieve CSPG inhibition were validated. Here, we demonstrate that after ventral root avulsion and immediate re-implantation, modulation of PTPσ by systemic delivery of ISP remarkably enhanced regeneration. ISP treatment reduced motoneuron death, increased the number of axons regenerating across scar tissue, rebuilt healthy neuromuscular junctions and enhanced motor functional recovery. Our study shows that modulation of PTPσ is a potential therapeutic strategy for root avulsion.

A root avulsion injury physically separates spinal nerves from the spinal cord, leading to severe disruption of the root itself as well as the associated spinal cord segment^1^. Avulsion injuries often occur in violent events, such as traffic and sports accidents or during a difficult childbirth^2^,^3^. Following root avulsion, there is rampant death of injured neurons, degeneration of axons, scar formation in the spinal cord and loss of synapses, which consequently result in disability of distal muscles and diminution of sensorimotor functions^1^,^3^,^4^. The avulsed spinal nerve root is made up of a longer distal segment of peripheral nerve and a small fragment of central nervous tissue, which normally forms a dome that protrudes a short distance into the nerve. The interface between the central nervous system (CNS) and peripheral nervous system (PNS) is known as the transitional zone (TZ)^1^,^5^. The CNS part contains high numbers of astrocytes which normally form channels within the basal lamina that allow the motor fibers to pass freely into the Schwann cell bands of Bungner. After avulsion injury, remaining astrocytes in the cord rearrange and hypertrophy to form a TZ scar which is similar to that found in a spinal cord injury^1^,^5^,^6^. In order to restore motor function after avulsion, injured motoneurons must survive and regenerate axons, which need to elongate through inhibitory scar tissue in the TZ before re-entering into the peripheral nerve trunk and eventually form synapses with distal target muscles^1^,^4^. Motoneuron survival and axonal regeneration can occur if the roots are surgically re-implanted, using proper techniques, onto the pia mater of the spinal cord close to the vicinity of the damaged ventral motoneuron pools^3^,^6^,^7^. However, axon regeneration and functional recovery are still quite unsatisfactory. Reactive astrocytes synthesize and secrete inhibitory chondroitin sulfate proteoglycans (CSPGs) into the extracellular matrix, which create a growth impediment^8^,^9^. Indeed, CSPGs have been known as the major inhibitor in scar tissue for years and digestion of lesion-induced CSPGs by Chondroitinase ABC (ChABC) enhanced axon regeneration and functional recovery after spinal cord injury^9^,^10^,^11^.

The mechanism of CSPG inhibition was not clear until the discovery of its neuronal receptor, protein tyrosine phosphatase-σ (PTPσ)^12^. Later, more receptors were identified, including a sister protein, leukocyte antigen-related protein (LAR) as well as the Nogo receptors 1 and 3^13^,^14^. Various CSPGs bind to the extracellular domain of PTPσ via their glycosaminoglycan (GAG) side chains^15^. Recent studies have shown that modulation of PTPσ could relieve axonal inhibition by CSPGs and enhance functional recovery after spinal cord injury^15^ and ischemic heart attack^16^.

Apart from exerting inhibition in the CNS, CSPGs also become prominently increased within the endoneurium in the injured distal peripheral nerve, which counterbalances regeneration-promoting Schwann cell derived factors, slowing regeneration^17^,^18^. Following sciatic nerve injury, regeneration was augmented by ChABC treatment^19^. Knockout of PTPσ accelerated axon extension and promoted functional recovery after peripheral nerve injury^20^.

Due to its important role in mediating CSPG inhibition, we hypothesized that PTPσ might be a potential therapeutic target for root avulsion injury. In the present study, we proposed that systemic manipulation of PTPσ by ISP^15^ could help axons navigate through scar tissue in the CNS-PNS TZ and promote functional restoration after avulsion injury. Therefore, we avulsed rat cervical ventral roots (C5, C6, and C7) on one side, followed with re-implantation of only the C6 root for potential regeneration. We demonstrate that sub-cutaneous ISP administration remarkably promoted motor functional recovery. We also report that the treatment rescued motorneurons from dying, increased axon regeneration and resulted in healthier synapses between motoneurons and muscle fibers. Our results highlight PTPσ as a promising therapeutic target for root avulsion injury.

## Results

### Motor functional recovery was enhanced by ISP treatment

**Figure 1:**
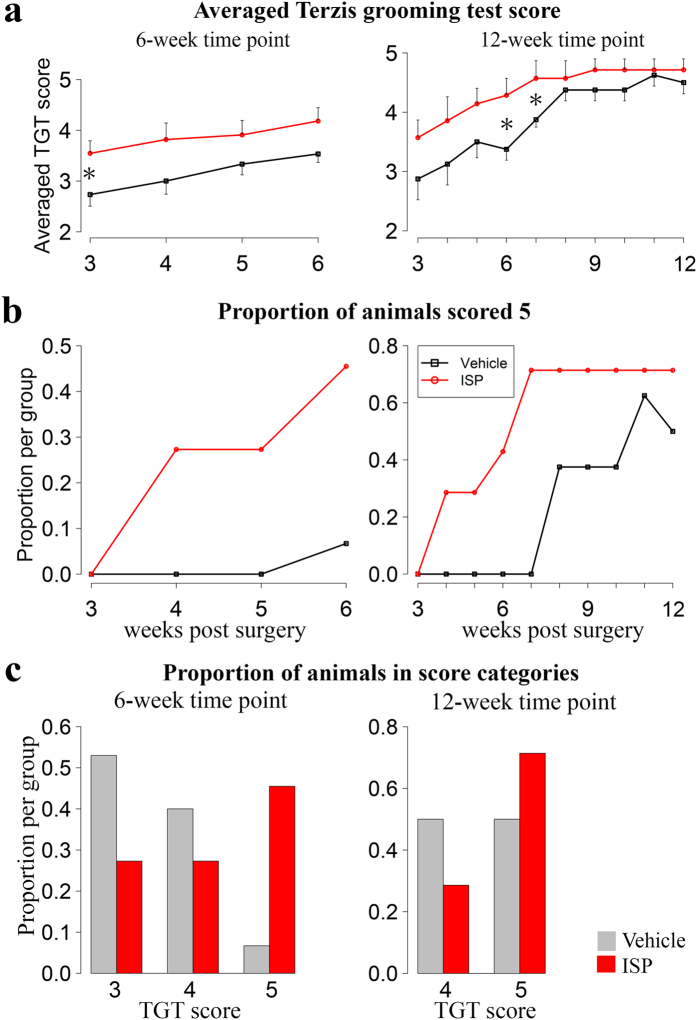
ISP treatment enhanced motor functional recovery after root avulsion injury/re-implantation repair, reflected by the Terzis grooming test. All left panels were from 6-week time point (n = 15 for vehicle, n = 11 for ISP), while right panel from 12-week time point (n = 8 for vehicle, n = 7 for ISP). (**a**) Averaged Terzis grooming test score was increased by ISP treatment. Data is expressed as mean + SEM for ISP group, or as mean-SEM for vehicle group. (*p < 0.05, Mann–Whitney U test). (**b**) Animals scored 5, indicating complete grooming recovery, accounted for higher percentage in ISP groups than vehicles. (**c**) Proportion of animals in each TGT score category at 6- and 12-week postoperatively. Vehicle rats had higher tendency to have a 3 or 4 score but less frequency to score 5.

Functional recovery is the ultimate aim in any therapy for root avulsion injury. Therefore, we first asked whether ISP enabled better motor functional recovery after spinal root avulsion injury/re-implantation surgery. The Terzis grooming test (TGT)^21^ evaluates motor function of the upper limb. A 0–5 rating criterion was utilized depending on the highest level to which the affected upper limb could reach. A zero score indicates loss of all function and 5 means complete grooming recovery. 24 hours after surgery, all animals were subjected to the Terzis grooming test and all scored 0, confirming successful surgery (Supplementary video 1). Functional recovery started within 3 weeks in both ISP and vehicle treated, re-implanted groups. However, averaged TGT scores were remarkably increased by ISP treatment, and significant differences were observed at 3-, 6- and 7-weeks post surgery (p < 0.05, Mann Whitney U test. Fig. 1a). At 6-weeks post surgery, 45.5% of ISP animals scored 5, while only 6.7% of vehicle animals achieved this level (Fig. 1b,c; Supplementary video 2, Supplementary video 3). The proportion of animals that scored 5 was always lower in the vehicle group throughout the testing period, even though they gradually recovered (Fig. 1b,c). At 12 weeks post surgery only 50% of vehicle treated rats scored 5, compared with 71.4% of ISP rats scoring 5 (Fig. 1b,c).

### Surviving and regenerating motoneurons were increased after ISP treatment

**Figure 2:**
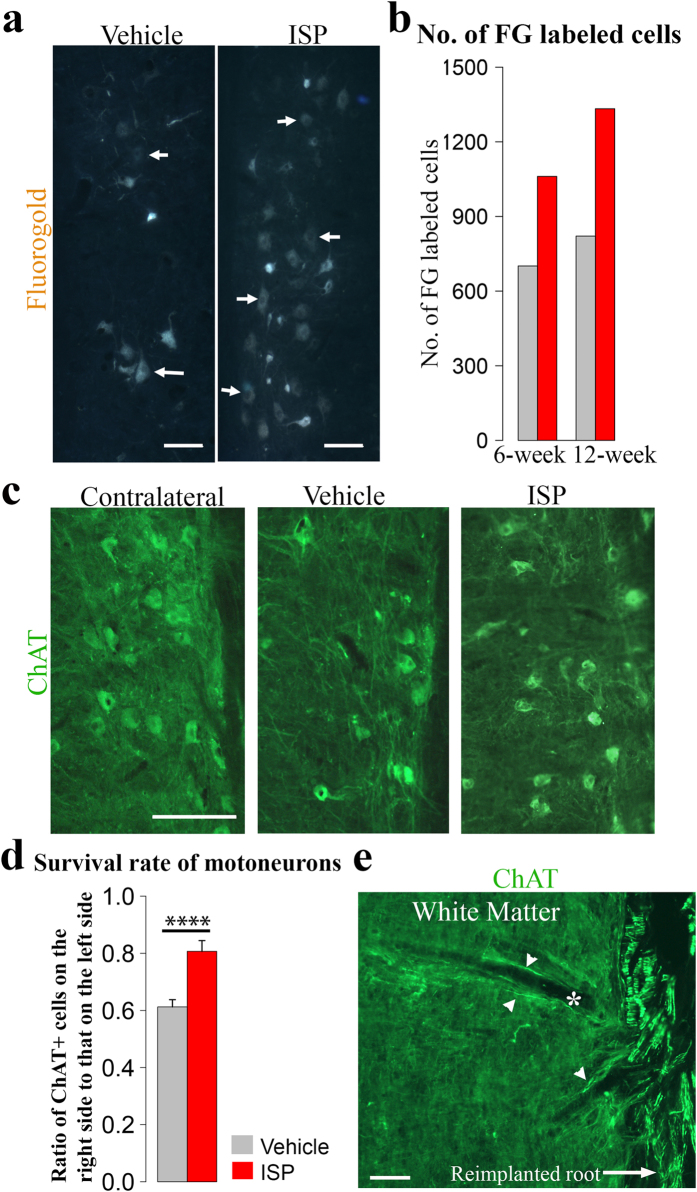
Motoneuron loss was reduced and regenerating cell number enlarged by ISP intervention. (**a**) Retrograde labeled cells located in C6-C7 spinal cord segments after injecting fluorogold into musculocutaneous nerve. More labeled cells were observed in ISP treated animals. (**b**) Averaged fluorogold labeled cell number at 6- and 12-week post surgery in both groups. At 6-week time point, 51.3% more cells were retrogradly labeled in ISP group than in vehicle rats, and this number was even enlarged to 61.5% at 12-week time point even though labeled cell number raised in both groups. (**c**) Representative images showing spinal cord sections (C6 and C7 segments) stained with ChAT antibody (green). Motoneuron loss after avulsion/re-implantation was reduced by ISP treatment. (**d**) Survival rate of motoneurons was calculated as the ratio of ChAT positive cell number in the ipsilateral spinal cord (C5-C7 segments) to that of the contralateral side. ISP treatment significantly increased survival rate of motoneurons from 61.2% in vehicle rats to 80.7% (Data expressed as mean ± SEM, n = 5, ****p < 0.0001, chi-square test). (**e**) Regenerating axons in ISP animals (stained with ChAT, green, white arrowhead), usually accompanied by a blood vessel (white asterisk), navigated directly across spinal white matter and the CNS-PNS TZ and formed physical connections with re-implanted spinal roots (white arrow). (Scale bars: 100 μm in (**a**,**e**), 200 μm in (**c**)).

We further asked whether the functional improvements were associated with more motoneurons regenerating axons, especially into the distal peripheral nerve. Therefore, we performed retrograde labeling studies by injecting fluorogold into the musculocutaneous nerve and counted labeled cells in the ventral spinal cord. The 2 rats with the best and worst Terzis grooming test scores were chosen from each group, at both 6- and 12-weeks post surgery. As observed at the 6-week time point, ISP rats had, on average, 51% more cells labeled retrogradely from the muscle. As regeneration proceeded, labeled cells increased in both groups. However, differences between the two treatment groups in labeled cell numbers was further enlarged at 12-week post surgery, with 62% higher amount of labeled cells in ISP animals (Fig. 2a,b). Next we asked whether the increased retrogradely labeled cell number could also be attributed to increased cell survival in addition to enhanced regeneration speed.

Since it is essential to maintain avulsed motoneurons alive in order to achieve functional restoration, we investigated whether ISP promoted a higher survival rate of motoneurons. Choline acetyltransferase (ChAT), as a marker of motoneurons, is transiently down-regulated after root avulsion injury but returns to detectable levels at 4 weeks postoperatively^22^. ChAT immunostaining has been used to quantify surviving motoneurons in long-term avulsion injury models^23^,^24^. Therefore, we selected 5 representative animals from each group and stained the 5^th^–7^th^ cervical segments of the spinal cord with ChAT antibodies. The survival rate of motoneurons is expressed as the ratio of the number of ChAT positive motoneurons on the ipsilateral side to that on the contralateral side. At 12-weeks post surgery, a significantly higher survival rate was detected in ISP animals compared with vehicle rats (p < 0.0001, chi-square test) (Fig. 2c,d). 61.2% of motoneurons remained alive in vehicle animals (which is consistent with our previous study of an avulsion-replantation model (62%)^7^). By contrast, ISP enabled survival of 80.7% motoneurons.

Importantly, sections of the cord near the graft/host interface gave the impression that ISP treatment enabled regenerating axons to navigate directly across the CNS-PNS TZ and to elongate into the re-implanted root (Fig. 2e). It is inferred that modulation of the CSPG receptor PTPσ provided a pro-regenerative environment for axon elongation across the spinal cord and TZ after root avulsion.

### ISP facilitated more axons with bigger sizes extending into the implanted nerve trunks

**Figure 3:**
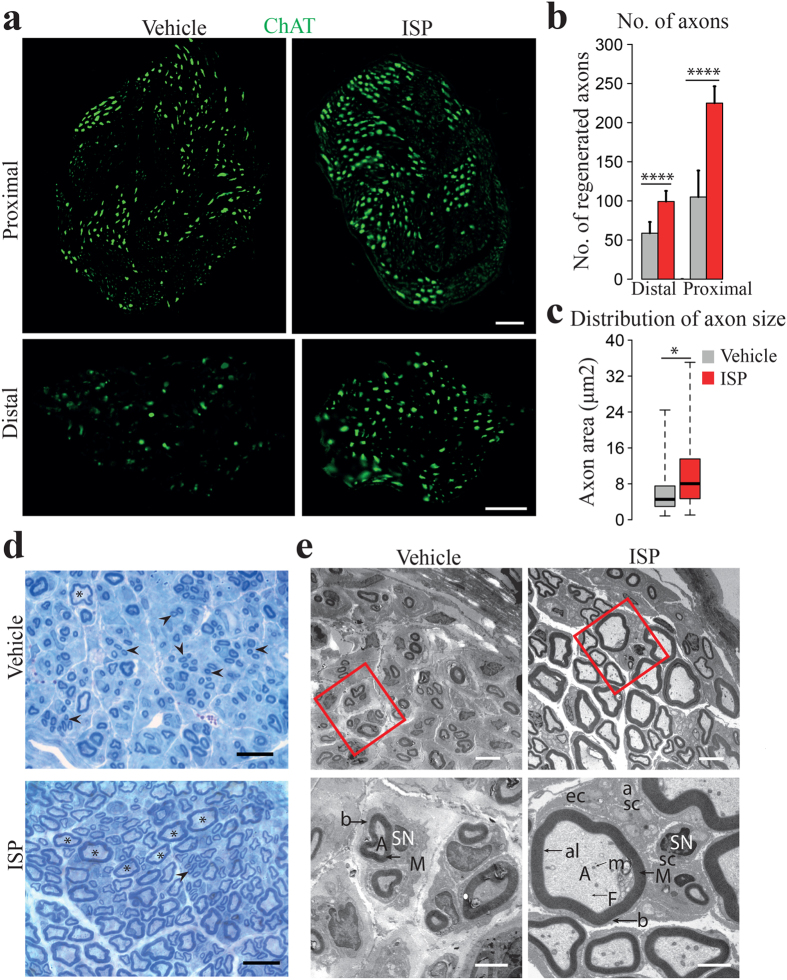
Numbers of motoneuron axons in the nerve trunk were increased and axon size enlarged in ISP rats. (**a**) Cross sections of proximal and distal musculocutaneous nerve (stained with ChAT, green). More axons were present at both sites in ISP animals. Vehicle treated rats showed not only less axon number, but also smaller size of axons with faint staining. (**b**) Numbers of axons at proximal and distal musculocutaneous nerve were significantly increased in ISP rats (Data expressed as mean ± SEM, ****p < 0.0001, chi-square test, n = 4). (**c**) Axonal size distribution of distal musculocutaneous nerve. Minimum, 1^st^ quartile, median, 3^rd^ quartile and maximum data points were marked in the plotting. 76.7% vehicle axons were smaller than 8 μm^2^, compared with only 50% of ISP axons in this size category. (*p < 0.05, student’s t test, n = 3). (**d**) Representative images of semithin sections of the distal musculocutaneous nerve show that ISP animals had more large-size axons while vehicle animals had more small-size axons. (Asterisks: axons bigger than 5 μm^2^; arrowhead: axons smaller than 5 μm^2^. (**e**) Electron micrographs of distal musculocutaneous nerve. ISP axons, clearly enwrapped within myelin sheaths by Schwann cells, were displayed with increased number and larger size. Areas in red rectangle are illustrated with higher magnification in lower panel with a counterclockwise rotation of 35°. (A: myelinated axon, a: unmyelinated axon; M: myelin sheath; SN: Schwann cell nucleus; al: axolemma; F: neurofilament; m: axon mitochondrion; b: basal lamina; sc: Schwann cell cytoplasm; ec: endonurial collagen fibers). (Scale bar: 50 μm in (**a**), 20 μm in (**d**), 5 μm in upper row of (**e**) and 2 μm in lower row).

We next confirmed quantitatively that ISP increased the number of motoneuron axons elongating into re-implanted injured nerves. Two sites along the musculocutaneous nerve were selected (proximal and distal end of the nerve) to count ChAT positive motoneuron axons. At both of the two sites, we detected twice the number of ChAT positive axons in ISP animals compared to the vehicle animals (Fig. 3a,b. p < 0.0001, chi-square test).

We further quantified axon size in the distal musculocutaneous nerve using semithin cross sections. About 23% of axons in the vehicle group were bigger than 8 μm^2^, whereas over 50% of ISP treated axons fell into the same size range (Fig. 3c,d. p < 0.05, student’s *t* test). Consistently, electron micrographs validated that ISP treatment enabled more axons with bigger sizes to extend through the distal musculocutaneous nerve. Ultrastructural examination showed comprehensive regeneration in ISP animals, in that axons with mitochondria and abundant neurofilaments were clearly ensheathed in myelin by Schwann cells and both basal lamina and endoneurium were well formed to pack single myelinated axons. Groups of small unmyelinated axons were embedded in non-myelinating Schwann cells (Fig. 3e, lower panel).

### ISP animals had healthier motor endplates and less muscle loss

**Figure 4:**
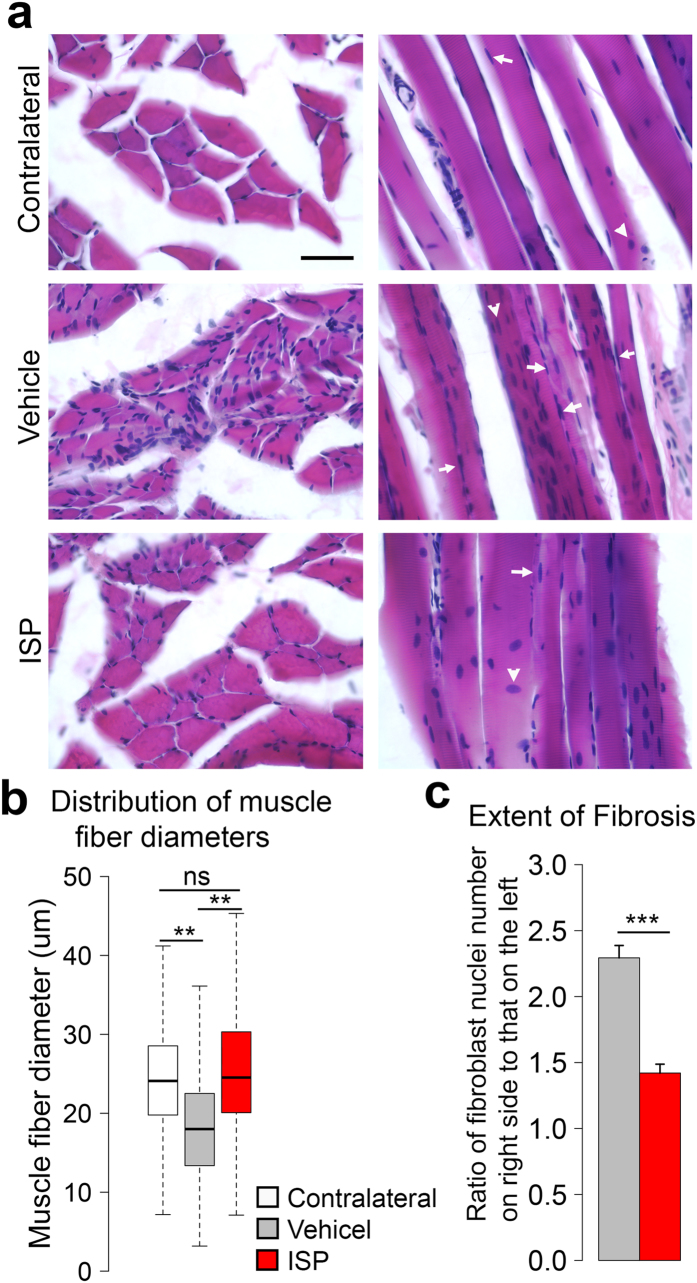
Muscle atrophy was reduced by ISP treatment. (**a**) Cross (left column) and longitudinal sections (right column) of biceps with H&E staining. Vehicle biceps exhibited more severe muscle atrophy, reflected by the shrunken sarcoplasm and presence of abundant fibroblast nuclei (white arrow). By contrast, ISP biceps with clear myocyte nucleus (white arrowhead) displayed less extent of fibrosis. (**b**) Distribution of muscle fiber diameter. Minimum, 1^st^ quartile, median, 3^rd^ quartile and maximum data points were plotted in the graph. 50% of contralateral and ISP muscle fibers had diameters bigger than 24 μm, while only 19% of vehicle muscle fibers showed the comparable diameters. (**p < 0.01, ns: not significant, z test, n = 6) (**c**) Extent of fibrosis was expressed as the ratio of fibroblast nuclei number in the ipsilateral biceps to that on the contralateral side. ISP treatment significantly reduced fibrosis. (Data presented as mean ± SEM, ***p < 0.001, student’s t test, n = 4; Scale bar: 50 μm in (**a**)).

We next evaluated recovery of the biceps, which is innervated only by the musculocutaneous nerve. Motoneuron axons exert a trophic effect on their controlled muscle fibers, via releasing neurotransmitter into the synaptic cleft and inducing contraction. Denervated muscle fibers develop morphological changes such as shrunken sarcoplasm and fibrosis^25^. As reinnervation proceeds, instead of being scattered as in the contralateral biceps, muscle fibers become clustered in groups. Because some motoneurons die and fail to influence their target myocytes again, these muscle fibers tend to become reinnervated by adjacent axon sprouts, which consequently lead to fiber type grouping^26^ (Fig. 4a, lower left). Vehicle muscle fibers exhibited smaller cross sectional areas and higher amounts of fibroblasts presented within the endomysium. By contrast, ISP muscle fibers with clear myocyte nuclei showed higher morphological similarity to normal ones (Fig. 4a). Indeed, 50% of muscle fibers from both contralateral and ISP biceps had diameters bigger than 24 μm, whereas only 19% of vehicle muscle fibers displayed comparable sizes (Fig. 4b). To quantify the extent of fibrosis, we counted the number of fibroblast nuclei in muscle fibers and calculated the ratio of these cells on the ipsilateral versus the contralateral sides. Fibrosis was significantly reduced by ISP administration (Fig. 4c, p < 0.001, student’s t test).

**Figure 5:**
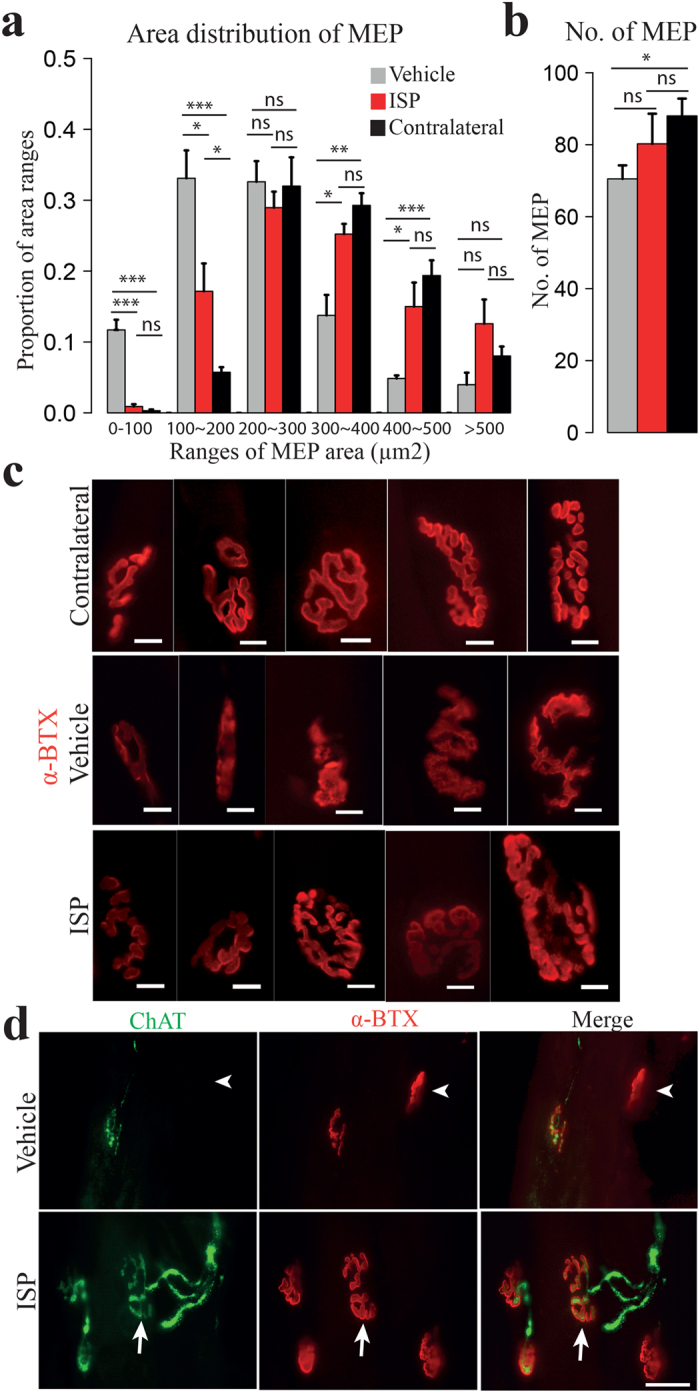
ISP facilitated healthier neuromuscular junctions. (**a**) Area distribution of MEPs. The size disribution of ISP MEPs displayed higher similarity to normal contralateral MEPs, whereas vehicle MEPs had higher tendency to fall into the small size ranges. (Data expressed as mean ± SEM, *p < 0.05, **p < 0.01, ***p < 0.001, ****p < 0.0001, ns: not significant, student’s t test, n = 4). (**b**) Averaged number of MEPs in biceps. Significantly smaller number was detected in vehicle biceps than that in the contralateral side, but no significant difference was found between ISP biceps and the un-operated side. (Data expressed as mean ± SEM, *p < 0.05, One-way ANOVA, n = 4). (**c**) Representative photos of motor endplates from contralateral, vehicle and ISP biceps. Vehicle MEPs not only have smaller sizes, but also show higher frequency to have ambiguous appearances and faint staining. (**d**) Illustrations showing motor endplates (stained with (α-BTX, red, middle panel) reinnervated by regenerated axons (stained with ChAT, green, left panel). Poor or lack of reinnervation (white arrowhead, upper panel) leaded to shrunken MEPs and vague morphology in vehicle animals. By contrast, ISP MEPs, with bigger sizes and clear shapes, showed nice co-staining with axon terminals (white arrow, lower panel) (Scale bar: 10 μm in (**c**), 20 μm in (**d**)).

The high similarity between contralateral (control) and ISP treated ipsilateral biceps was further confirmed by motor endplate (MEP) assessment. MEPs are referred to as postsynaptic folds of neuromuscular junctions, in which acetylcholine receptors (AchRs) are located in high density. To assess the number and size changes of motor endplates, we stained the muscle sections with α-bungarotoxin (α-BTX), which specifically binds to AchRs. Numbers of MEPs were significantly decreased in vehicle biceps, compared with contralateral muscle, whereas numbers of MEPs in ISP biceps showed no obvious difference with normal ones (Fig. 5b). By randomly measuring areas of 150–200 MEPs from each biceps, we found that the area distribution of ISP MEPs showed higher similarity to normal ones, whereas Vehicle MEPs tended to fall into smaller size categories (Fig. 5a,c), indicating loss of AchR area. Finally, vehicle motor endplates had a higher tendency to be faintly stained, suggesting lower density of AchRs that might result from diminished reinnervation (Fig. 5d, upper panel). Indeed, MEPs in ISP treated animals were well infiltrated by axon terminals, displayed bigger sizes and had clearer morphology (Fig. 5d, lower panel). Shunken MEPs with fewer AchRs appeared in the biceps of vehicle treated animals, which suggested that ISP promoted healthier neuromuscular junctions.

### Improved muscle recovery was associated with electrophysiologically healthier motor units

Enhanced functional and morphological muscle recoveries were also reflected by improvements in the physiology of reinnervated motor units in ISP treated animals. Needle electromyography (EMG) detects extracellular electrical activity of muscle fibers and is applied to assess the health of motor units clinically. Denervated muscle fibers become supersensitive, which is reflected by the existence of spontaneous potentials during the resting state. These include fibrillation potentials and fasciculations^27^. Fibrillations can be caused by hypersensitivity of muscle fibers to acetylcholine^28^ or partial depolarization and spontaneous oscillations of the membrane potential due to altered Na channel density and kinetics^29^. Fibrillations persist for months after nerve lesion but they gradually disappear as reinnervation progresses. Clinically, the number of sites and the frequency of spontaneous potentials are adopted as rating criteria to evaluate the changes^27^.

**Figure 6:**
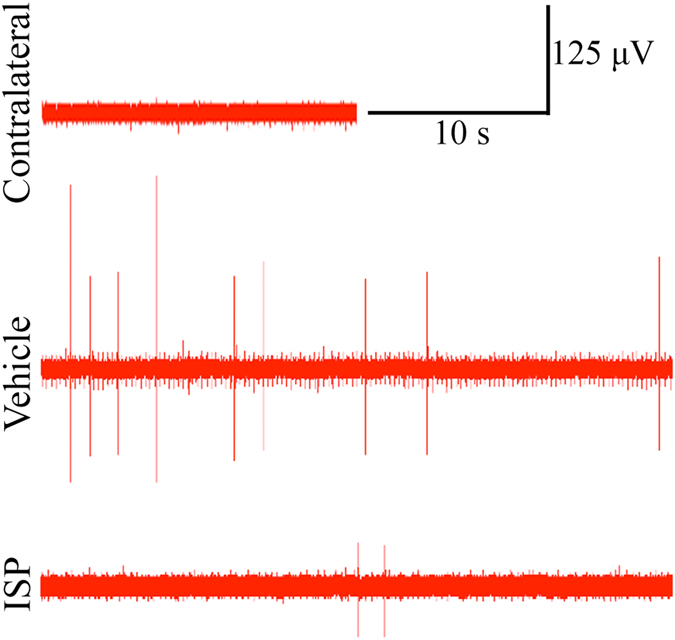
Reduced extent of electromyography abnormalities were recorded in the ISP treatment group. Representative traces of resting potentials are shown. Fibrillations resulted from denervated motor units were not detected in contralateral biceps (upper panel) but appeared in both vehicle (middle panel) and ISP (lower panel) rats.

Animals with different TGT scores from the two treatment groups were subjected to needle EMG at 12-week postoperatively (Table 1). No spontaneous responses were detected in the biceps contralateral to the lesion side (Fig. 6, upper row). However, fibrillations appeared in the ipsilateral biceps of both vehicle (Fig. 6, middle row) and ISP treated (Fig. 6, lower row) animals. Resting potentials were recorded in 4–8 sites in each biceps and the number of spontaneous potentials counted. In line with the behavioral test, animals with better TGT performance had fewer sites displaying spontaneous potentials, and the frequency of the potentials decreased accordingly. (Fig. 6, Table 1).

## Discussion

When a root avulsion injury occurs, the rupture site tends to localize in the CNS-PNS TZ^30^. Thus, spinal root avulsion injury is unusual in that it affects both PNS and CNS tissues simultaneously. In addition, a large proportion of affected motoneurons eventually die^7^. This is likely due to the close proximity of the lesion to the cell body. Pioneering studies by Carlstedt^31^,^32^ showed that following avulsion, motor axon regeneration (often as sprouts from dendrites^33^) could occur after root re-implantation, although regenerated axons often took meandering courses in the lateral white matter, rather than projecting directly into replanted roots^34^. Also, the suboptimal ability of axons to bypass the TZ coupled with the long distances and limited elongation speed due to CSPG accumulation along the length of the re-implanted nerve trunk^18^, together lead to prolonged and inefficient regeneration. All of these factors contribute to atrophy of distal muscle and loss of motor functions after avulsion injury even with re-implantation. In our rat model of avulsion injury + re-implantation repair, significant motor functional differences between the vehicle and ISP treated groups were observed in the first 7 weeks after surgery. This suggests that while axonal regeneration occurs in both ISP and vehicle treated animals, ISP treatment could increase the speed of regeneration. ISP might have higher therapeutic potential in larger animals, such as primates, where axons need to re-elongate longer distances before they could innervate their target muscles.

In responding to root avulsion injury, abundant astrocytes in the CNS-PNS transitional zone are activated and synthesize CSPGs in hours, depositing them in extracellular matrix of the scar. Indeed, after many types of CNS and PNS injuries CSPGs accumulate rapidly and last for months^9^,^35^. Many studies have demonstrated that suppression of CSPG inhibition by administration of chondroitinase enhanced axon regrowth. However, this approach mandates that the enzyme be directly injected into the zone of interest, which can cause further damage^36^. The identification of neuronal CSPG receptors has now made it possible to manipulate CSPGs-axon interactions globally via the use of tissue penetrating TAT-peptides. Indeed, systemically blocking PTPσ using a small peptide ISP, has allowed sprouting and regeneration of axons after SCI and heart attack, producing robust functional recovery^15^,^16^. We now report that after spinal root avulsion injury and re-implantation repair, using the same peptide to relieve CSPG inhibition, we could remarkably promote regeneration, resulting in 1) increased survival rate of injured motoneurons; 2) enhanced axonal regrowth across inhibitory CNS scar into the re-implanted spinal roots; 3) more regenerated axons with enlarged size in the peripheral nerve trunk; 4) decreased muscle atrophy and 5) more rapid motor functional recovery accompanied by reductions in electromyography abnormalities. Thus, local systemic delivery of ISP successfully released PTPσ mediated CSPG inhibition on axon outgrowth and enhanced functional return.

Severe progressive neuron death following a root avulsion injury^3^,^4^ is a major impediment to recovery. There is evidence that avulsed motoneurons rapidly enter an apoptotic state as they upregulate nitric oxide synthase (nNOS) and those with nNOS expression eventually die^4^,^37^. Indeed, 12 weeks after injury, only 14% of lesioned motoneurons survive and while the numbers of surviving cells can be increased to 62% by immediate root re-implantation^7^, the surgical procedure alone is not sufficient to rescue enough motoneurons. Application of neurotrophic factors such as glial cell line-derived neurotrophic factor (GDNF) and brain-derived neurotrophic factor (BDNF)^38^,^39^ were beneficial to motoneuron survival. However, there were also reports that neuroma-like structures formed several months after GDNF overexpression^39^ and high levels of neurotrophic factors prevented directional axon outgrowth into implanted roots^24^. In the present study, we found that via overcoming a regeneration barrier, ISP treatment enabled 80% of motoneurons to survive at 12 weeks after injury with many more regenerating into the implant. It can be inferred that a pro-regenerative environment might benefit motoneuron survival. However, whether PTPσ is directly involved in motoneuron survival is still unknown.

With its up-regulation in the endoneurium following peripheral nerve injury, CSPGs counterbalance the pro-regeneration factors in the Schwann cells and their enveloping basal laminae^17^. Indeed, peripheral axon elongation was accelerated in short nerve grafts by chondroitinase pre-digestion of CSPG^18^. Following a root avulsion injury, the distal peripheral nerve experiences degeneration and CSPG is accumulated along the full extent of the endoneurium. However, the long length of peripheral nerves makes it difficult to effectively treat with topically applied chondroitinase. Importantly, genetic knockout of PTPσ enhanced axon extension and promoted functional recovery after crushing peripheral nerve injury^20^,^40^. Thus, in addition to modulating CSPG mediated inhibition at the TZ, the peptide is also likely serving to modulate CSPG-axon interactions all along the route of regeneration approaching the denervated muscles.

We noticed that target reinnervation was improved by ISP treatment, which was mirrored by reduced muscle atrophy, as well as more re-innervated muscle units and healthier motor endplates. After nerve lesion, regenerated motor axons commonly re-innervate their original neuromuscular junctions^41^. However, synapse remodeling via sprouts from adjacent axons also exists, and the potential extent of such plasticity depends on the magnitude of the injury^42^,^43^. We deliberately did not re-implant the C5 and C7 roots to minimize the amount of potential axonal sprouting from spared fibers. Thus, most of the muscle re-innervation was forced to come from re-implanted C6. Rich and Lichtman^44^ illustrated that a higher percentage of shrunken motor endplates develops after transection/re-anastomosis injury compared to crush injury and delayed reinnervation resulted in a loss of AchR area in the postsynaptic zones. Indeed, we also found that after root avulsion + re-implantation, AchR areas shifted to smaller ranges in vehicle rats, and loss of AchRs in motor endplates contributed to their faint staining and ambiguous appearance. In contrast, the area distribution of ISP MEPs displayed high similarity to normal ones and it was surprising that the number of MEPs in ISP treated biceps showed no significant differences from normal muscle. This occurred even though C5 and C7 were not potential regeneration conduits. This suggests that the ramification abilities of the limited numbers of regenerating axon terminals at their muscle targets may have been exuberant.

Besides its function in axonal outgrowth and guidance, PTPσ is also identified as a synapse organizing protein in development^45^. Together with other IIa receptor-type protein tyrosine phosphatases (RPTPs) such as LAR and PTPδ, PTPσ is involved in mediating presynaptic differentiation and triggering postsynaptic differentiation by participating in extracellular adhesion and intracellular signaling^45^,^46^,^47^. Indeed, recent studies have shown that PTPσ is an essential player in excitatory synapse formation by interacting with postsynaptic NGL-3^48^ and Slitrks^49^. In addition, excitatory synapse transmission requires binding of PTPσ to the glypican-4/LRRTM4 complex^50^. It would be interesting to see whether and how PTPσ is involved in the formation, maintenance and remodeling of neuromuscular junctions.

In summary, we demonstrated that modulation of the CSPG receptor PTPσ by ISP facilitated regeneration after root avulsion injury and promoted motor functional recovery in rats. Our work highlighted PTPσ as a promising therapeutic target in treating root avulsion injury but also perhaps in a variety of other PNS indications where trauma or disease related scarring leads to regenerative failure.

## Materials and Methods

### Animals

Adult female Sprague Dawley rats (2–3 months old, 200–300 g body weight) were ordered from Laboratory Animal Unit of the University of Hong Kong. The university Committee for Use of Live Animals in Teaching and Research approved all animal handling and operation procedures. All experiments were carried out in accordance with the approved guidelines.

### Avulsion-replantation injury model

Animals were anaesthetized with a mixture of ketamine (80 mg per kg of body weight) and xylazine (8 mg per kg), by intraperitoneal injection. The right spine segments from the 4th cervical (C4) to the 2nd thoracic (T2) were exposed and a dorsal laminectomy was performed on laminae C4 to C7. After opening the dura matter, the right side C5-C7 roots (both dorsal and ventral) were avulsed using a fine glass hook. The avulsed C5 and C7 roots together with their connected spinal nerves were cut and removed, leaving a gap between the nerves and spinal cord to prevent regrowth, whereas the ventral roots of C6 were reattached back to spinal cord for regeneration, following procedures described previously^7^. Any injury to the spinal cord was avoided.

### Treatment and grouping

ISP was designed and provided by Jerry Silver’s lab. The dosage and application of ISP was empirically determined by previous work on contusive spinal cord injury^15^. Started at 24 hours post surgery, all 26 injured rats were treated daily with ISP (500 μl at concentration of 5 μM, 11 μg/day) or vehicle (5% DMSO in saline, 500 μl), by subcutaneous injection near the injury site. For ISP administrated animals, 4 were randomly chosen for 6-week time point analyses, and the other 7 rats were kept for 12 weeks until the endpoint. For the vehicle group, 7 rats were utilized for the 6-week time point study and 8 rats for the 12-week time point.

### Behavioral testing

The Terzis grooming test^21^ (TGT) was conducted to evaluate motor function of the upper limb. Around 5 ml water was gradually sprayed onto the rat snout using a 5 ml syringe, which would elicit bilateral grooming responses by the upper limbs. Pre-operative screening for the grooming test was performed in randomly selected animals and all scored 5 on both left and right sides. A 0–5 rating criterion was applied for the ipsilateral upper limb: 0, no response; 1, flexion at elbow, not reaching the snout; 2, flexion reaching the snout; 3, reaching below the eyes; 4, reaching to the eyes; 5, reaching to the ears and beyond. 24 hours after surgery, TGT was performed and successful operations were indicated by a 0 score. From the 3^rd^ week post surgery until the endpoint, animals were subject to weekly TGT to monitor motor functional recovery. Data was shown as mean ± SEM. Mann–Whitney U test was applied to perform statistical analysis.

### Retrograde labeling and labeled cell number counting

2 animals with the best and worst TGT performance were selected from each group at the 6- and 12-week time points to perform retrograde labeling studies. Around 0.8 μl of Fluorogold (Fluorochrome, 6% in sterilized water) was slowly injected into the ipsilateral musculocutaneous nerve. 4 days after injection, labeled animals were perfused and fixed with 4% paraformaldehyde in phosphorylated buffer (PB). Spinal cord segments C5-T1 were collected and sectioned longitudinally, at a thickness of 25 μm. Section observation was carried out under microscopy (Zeiss Axiphot Microscope) with a 420 nm filter. Labeled cells in the C5-C7 segment were counted on every other section.

### Assessment of motoneuron survival rate

Fixed spinal cord segments C5-T1 were sectioned at a thickness of 25 μm. After antigen retrieval by incubation with 10 μg/ml proteinase K at 37 °C for 10 minutes, every third section was stained with a ChAT antibody (Millipore, 1:100) overnight at 4 °C, followed with a secondary antibody (Invitrogen, 1:400, conjugated to AlexaFluor 568) incubation for 1.5 hour at room temperature. Numbers of ChAT positive motoneurons were counted separately for the ipsilateral and contralateral sides. The ratio of operated side number to that of the intact side was computed. Data was displayed as mean ± SEM with chi-square test for statistical analysis.

### Motor endplate assessment

Fixed ipsilateral and contralateral biceps (from 12-week time point rats) were sectioned longitudinally from the ventromedial to superficial side, at a thickness of 14 μm. Every 4th section was collected and 20 slides for each muscle were harvested to stain with α-Bungarotoxin (α-BTX, Invitrogen, Alexa Fluor 594 conjugated, 1:500) for 30 minutes. Numbers of motor endplates (MEP) on each section were counted. 10–15 MEPs from each section and 150–200 MEPs from one muscle were randomly chosen to take photos (MBF Nikon Microscope). MEP area was measured using image J. Area range was divided into six categories: from 0 to 500 μm^2^ (separated by hundreds) and over 500 μm^2^. For every biceps, numbers of MEPs falling into each category were counted and the proportion to the total number calculated. Data was expressed as mean ± SEM. One-way ANOVA was used for MEP number assessment, while student’s t test was applied for MEP area distribution analysis.

### Measuring axon number in the nerve trunks

Two sites along the musculocutaneous nerve (distal to lateral cord and 2 mm proximal to biceps) were selected to assess numbers of motoneuron axons. Cross sections were cut at a thickness of 3 μm and immunostaining with ChAT was carried out as described in assessment of motoneuron survival rate. The number of ChAT positive axons was counted at each site. Data was demonstrated as mean ± SEM, and chi-square test was applied for statistical analysis.

### Electron microscopy

Two sites along the musculocutaneous nerve (distal to lateral cord and 2 mm proximal to biceps) were fixed overnight with a mixture of 2% PFA and 2.5% glutaraldehyde in 0.1 M PB, followed with overnight fixation using 1% osmium tetroxide. The tissue was then dehydrated in graded ethanol (30%, 50%, 70%, 80%, 90%, 95% for 5 minutes each and 100% for 3 times in 30 minutes) and infiltrated with propylene oxide (PO) twice in 30 minutes, PO: Epon (1:1) for one hour and pure Epon overnight. After that, the nerve was embedded in Epon and polymerized at 60 °C for 72 hours. Semithin sections (0.5 μm) were cut by a microtome (Ultracut) using a glass knife before staining with 0.5% toluidine blue in 1% borax for 35 s. A light microscope (MBF Nikon Microscope) was used for observation and images were digitalized. Around 80 axons from each animal were randomly selected for area measurement using image J. Student’s *t* test was applied for statistical analysis. Ultrathin sections of 90 nm thickness were stained with 3% uranyl acetate and 1% lead citrate and digital images captured by electron microscopy (TEM, Phillip model 208).

### Hematoxylin and Eosin (H&E) staining, muscle fiber diameter measurements and fibrosis assessment

Cross and longitudinal sections of biceps were cut and processed for H&E staining. Briefly, sections were deparaffinized by toluene and rehydrated by degraded ethanol (100%, 100%, 95%, 95%, 75% ethanol, 2 minutes each). Nuclei were stained with Harris haematoxylin for 8 minutes, followed by differentiation with 0.3% acid alcohol. Cytoplasm was stained with eosin for 2 minutes. Sections were then subject to dehydration by graded ethanol (75%, 95%, 95% and 100%, 2 dips each; 100% for 2 minutes; 100% for 12 minutes). Light microscopy (MBF Nikon Microscope) was used for observation and images were digitalized. Around 200 muscle fibers from each biceps were randomly selected for diameter assessment. Z test was applied for statistical analysis. 10 photos were randomly digitized from each biceps and fibroblast nuclei on each photo were quantified. The ratio of the number of fibroblast nuclei in the ipsilateral biceps to that on the contralateral side was computed. The data was expressed as mean ± SEM with a student’s t test as statistical analysis.

### Electromyography

12 weeks post surgery, 2 animals (1 scored 5 in Tersiz grooming test and the other graded 4) from both vehicle and ISP groups were selected for needle electromyography (EMG) analysis using an RM6240 multichannel signal process system (Chengdu Instrument Factory). Animals were anaesthetized and both left and right biceps were exposed, as well as their connected musculocutaneous nerves. The nerve was hooked onto a stimulation electrode, while two recording needle electrodes were inserted into the biceps at a 1–2 mm depth and with 5–7 mm distance. At least 4 different locations in one biceps were chosen to test the electrical activity of different motor units. Resting potential was recorded for around 2 minutes for each location, then different voltage stimulations (from 0 to 2 mV) were applied and responses recorded.

### Data analysis

Data expression and methods of hypothesis testing were described separately in each section. Student’s *t* test was performed using Microsoft Excel. Other testing and all plotting were done using R. Significant difference was considered when P value was less than 0.05. All experiments were performed in a blinded fashion.

## Additional Information

**How to cite this article**: Li, H. *et al*. Enhanced regeneration and functional recovery after spinal root avulsion by manipulation of the proteoglycan receptor PTPσ. *Sci. Rep*. **5**, 14923; doi: 10.1038/srep14923 (2015).

## Supplementary Material

Supplementary Video S1

Supplementary Video S2

Supplementary Video S3

## Figures and Tables

**Table 1:** Spontaneous potential detected by needle EMG.

Treatment	TGT score	Sites tested	Sites with spontaneous potential	Frequency of spontaneous potential (Hz)
Vehicle	4	5	5	0.272
	5	4	1	0.109
ISP	4	5	3	0.210
	5	8	2	0.069
